# CRISPR-Cas-mediated unfolded protein response control for enhancing plant stress resistance

**DOI:** 10.3389/fpls.2023.1271368

**Published:** 2023-10-16

**Authors:** Bich Ngoc Vu, Tien Van Vu, Jae Yong Yoo, Ngan Thi Nguyen, Ki Seong Ko, Jae-Yean Kim, Kyun Oh Lee

**Affiliations:** ^1^ Plant Molecular Biology and Biotechnology Research Center (PMBBRC), Gyeongsang National University, Jinju, Republic of Korea; ^2^ Division of Applied Life Science (BK21 Four), Gyeongsang National University, Jinju, Republic of Korea; ^3^ Nulla Bio Inc., Jinju, Republic of Korea

**Keywords:** endoplasmic reticulum (ER) stress, unfolded protein response (UPR), genome editing, CRISPR-Cas, crop improvement

## Abstract

Plants consistently encounter environmental stresses that negatively affect their growth and development. To mitigate these challenges, plants have developed a range of adaptive strategies, including the unfolded protein response (UPR), which enables them to manage endoplasmic reticulum (ER) stress resulting from various adverse conditions. The CRISPR-Cas system has emerged as a powerful tool for plant biotechnology, with the potential to improve plant tolerance and resistance to biotic and abiotic stresses, as well as enhance crop productivity and quality by targeting specific genes, including those related to the UPR. This review highlights recent advancements in UPR signaling pathways and CRISPR-Cas technology, with a particular focus on the use of CRISPR-Cas in studying plant UPR. We also explore prospective applications of CRISPR-Cas in engineering UPR-related genes for crop improvement. The integration of CRISPR-Cas technology into plant biotechnology holds the promise to revolutionize agriculture by producing crops with enhanced resistance to environmental stresses, increased productivity, and improved quality traits.

## Introduction

The endoplasmic reticulum (ER) is a crucial organelle responsible for protein folding and modifications in eukaryotic cells (Schuldiner and Schwappach, 2013). Proper protein folding is essential for the proper function of secretory and membrane proteins, which account for approximately 30% of the total proteome (Wallin and Von Heijne, 1998; Schubert et al., 2000). Various post-translational modifications, such as N-linked glycosylation, disulfide bond formation, and chaperone-assisted folding, occur in the ER lumen to ensure proper protein folding (Duwi Fanata et al., 2013). However, protein folding, occurring within the ER, can be disrupted not only by internal factors, such as genetic mutations and hereditary metabolic defects, but also by external factors, such as biotic and abiotic stresses (Park and Park, 2019). When proteins fail to fold properly or become misfolded due to these intrinsic or extrinsic factors, their accumulation within the ER leads to an aberrant cellular condition known as ER stress (Howell, 2013). To address ER stress, eukaryotic cells activate a series of compensatory adaptive mechanisms, collectively called the unfolded protein response (UPR) (Kozutsumi et al., 1988; Harding et al., 2002). The UPR activates a process that increases the expression of ER chaperone genes, enhancing the protein folding capacity of the ER, while also inhibiting protein synthesis and promoting ER-associated protein degradation (ERAD) to alleviate the burden of misfolded proteins (Duwi Fanata et al., 2013). When the UPR is not able to mitigate ER stress, it can lead to apoptosis or cell death, which may contribute to the development of prominent stress-related phenotypes, such as inhibited growth or developmental abnormalities (Hetz, 2012; Angelos et al., 2017).

Numerous studies have reported on the evolutionarily conserved UPR mechanism in eukaryotes, from yeast to animals and plants (Chakraborty et al., 2016; Howell, 2021). The UPR has been extensively characterized in mammals, where it consists of three ER signaling pathways: activating transcription factor 6 (ATF6), inositol requiring enzyme 1 (IRE1)-mediated splicing activation of X-box binding protein 1 (XBP1) mRNA, and double-stranded RNA-activated protein kinase (PKR)-like endoplasmic reticulum kinase (PERK) (Chakrabarti et al., 2011). Aberrant UPR has been implicated in a wide range of disease states, including diabetes, immune and inflammatory disorders, and cancers (Marciniak, 2019). Thus, signaling pathways of the UPR have emerged as a potential therapeutic axis for treating various diseases (Marciniak, 2019). While UPR mechanisms in mammals have been a subject of extensive research, exploration into the molecular mechanisms of ER stress responses in plants began more recently. Early investigations into plant UPR primarily started in the early 2000s, with a surge of substantial research outputs emerging a decade later (Koizumi et al., 2001; Noh et al., 2002; Deng et al., 2011; Nagashima et al., 2011; Moreno et al., 2012). With the advent of omics technologies, the field of plant UPR research is now transitioning into a new era characterized by big data. Two ER stress-transducing pathways have been identified in plants: IRE1a and b, which are functional homologs of IRE1 in mammals, and basic leucine zipper protein 17 and 28 (bZIP17 and bZIP28), which are functional homologs of ATF6 in mammals (Kim et al., 2022a). These pathways are involved in the UPR and perform similar functions to their counterparts in mammals. However, the existence of the PERK branch in plants, which is present in mammalian cells, is still unknown (Bao and Howell, 2017).

In sessile plants, the inherent inability to evade unfavorable environmental conditions results in frequent exposure to various abiotic and biotic stresses, such as drought, temperature fluctuations, salinity, herbicidal exposure, and pathogen infection (Park and Park, 2019). These stresses detrimentally impact crop yields, posing significant challenges to global food security. Furthermore, climate change-induced alterations in pathogen and insect behavior contribute to substantial reductions in crop productivity worldwide (Anderegg et al., 2020; Hassani et al., 2020; Van Houtan et al., 2021; Von Der Gathen et al., 2021; Zandalinas et al., 2021). Therefore, it is important to better understand the mechanisms underlying the impacts of these stresses on various crops. This knowledge will facilitate the optimization of tolerance and resistance to both biotic and abiotic stresses, and will ultimately contribute to the optimization of plant growth, development, yield, and quality (Rivero et al., 2022).

In recent years, CRISPR-Cas-based precise genome editing has emerged as a powerful tool, enabling the study of molecular mechanisms associated with ER stress and crop improvements (Singh et al., 2019; Um et al., 2021). CRISPR-Cas9, initially discovered in bacteria, has been engineered for use in various plant species to improve yield, quality, and stress tolerance (El-Mounadi et al., 2020; Vu et al., 2020a). There are several prospective strategies in which the CRISPR-Cas-based genome editing technology can be applied to UPR research. For instance, researchers can employ CRISPR-Cas to knockout or knockdown UPR-related genes. By investigating how these engineered plants respond to ER stress and the phenotypes they exhibit, scientists can gain valuable insights into the role of the targeted genes in the UPR pathway (Mishiba et al., 2019; Liu et al., 2020). Furthermore, the CRISPR-Cas system holds potential for modifying *cis*-regulatory elements or promoter regions in the genome, which in turn control gene expression, to augment stress resilience and other desirable traits (Lim et al., 2022). In this review, we also discuss several promising applications and future prospects of employing the CRISPR-Cas-based genome editing technology for strategic modifications of genes associated with ER stress responses, aiming to improve stress tolerance, productivity, and crop quality.

### UPR in plant adaptation to biotic and abiotic stresses

Plant adaptation to environmental stress is a complex process that involves a range of molecular, physiological, and biochemical responses. In plant stress response research, the majority of investigations have focused on single biotic or abiotic elements; however, the simultaneous presence of both biotic and abiotic stresses can markedly influence plant growth, productivity, and viability (Park and Park, 2019). To cope with these multiple stresses, plants initiate a range of signaling pathways and regulatory processes to preserve homeostasis and adapt to changing environmental conditions. The UPR is one such mechanism; it is a conserved response found across eukaryotic organisms, including plants, and plays a critical role in cellular adaptation to stress (Chakraborty et al., 2016). The UPR has emerged as a crucial regulatory mechanism in plant adaptation to combined biotic and abiotic stresses, allowing plants to cope with the challenges posed by their environment.

Under heat stress conditions, protein folding becomes perturbed, and several ER membrane-associated transcription factors relay stress signals to the nucleus, which in turn activates stress-responsive genes (Fragkostefanakis et al., 2016; Reyes-Impellizzeri and Moreno, 2021). It has been discovered that in diverse plant species, such as *Arabidopsis thaliana* (Arabidopsis) and *Zea mays* (maize), heat stimulation causes IRE1 to splice *bZIP60* mRNA (Deng et al., 2011; Li et al., 2012; Neill et al., 2019). The expression of active bZIP60 also elevates the transcription of heat shock protein (HSP) genes, suggesting a link between the UPR and the heat shock response mechanism (Li et al., 2020c). The transcription factor bZIP28 regulates the expression of UPR-related genes in response to heat stress conditions via a proteolytic mechanism, which triggers the translocation of bZIP28 to the nucleus (Iwata et al., 2017). Drought and salt significantly impact plant development and yield. In response to salt stress, bZIP17 is cleaved by site-1 proteases (S1P) and translocated to the nucleus to activate UPR genes (Liu et al., 2007b). It has been shown that the transcription factors bZIP60 and bZIP17 orchestrate the expression of the molecular chaperone gene, luminal-binding protein 3 (BiP3), as well as several genes implicated in the response to salt stress conditions (Henriquez-Valencia et al., 2015). Elevated expression of BiP has been observed to augment drought tolerance in a variety of plant species, such as *Glycine max* (soybean), *Nicotiana tabacum* (tobacco), and Arabidopsis (Coutinho et al., 2019). Infection by pathogens has been shown to induce ER stress in plants, with the IRE1-bZIP60 signaling pathway playing a crucial role in mounting a defense against the fungal pathogen, *Alternaria alternate* (Xu et al., 2019). Plants with mutations in *IRE1* and *bZIP60* are more susceptible to bacterial and viral infections (Moreno et al., 2012). In *Nicotiana benthamiana*, UPR was activated by the Geminivirus satellite-encoded βC1, which induces the nuclear export of NbbZIP60 to evade the plant defense response (Zhang et al., 2023). Overall, these findings indicate that various biotic and abiotic factors can disrupt protein folding capacity and activate the UPR in plants. As environmental stresses continue to impact global agriculture, the role of the UPR in facilitating plant adaptation to combined biotic and abiotic stresses is becoming a more significant area of research. Investigating the function of the UPR in plants has the potential to enhance crop improvement and sustainable agriculture practices, making the study of UPR activation in response to stress in plants increasingly important for agricultural research.

## UPR pathways in plant cells: mechanisms and regulation

**Figure 1:**
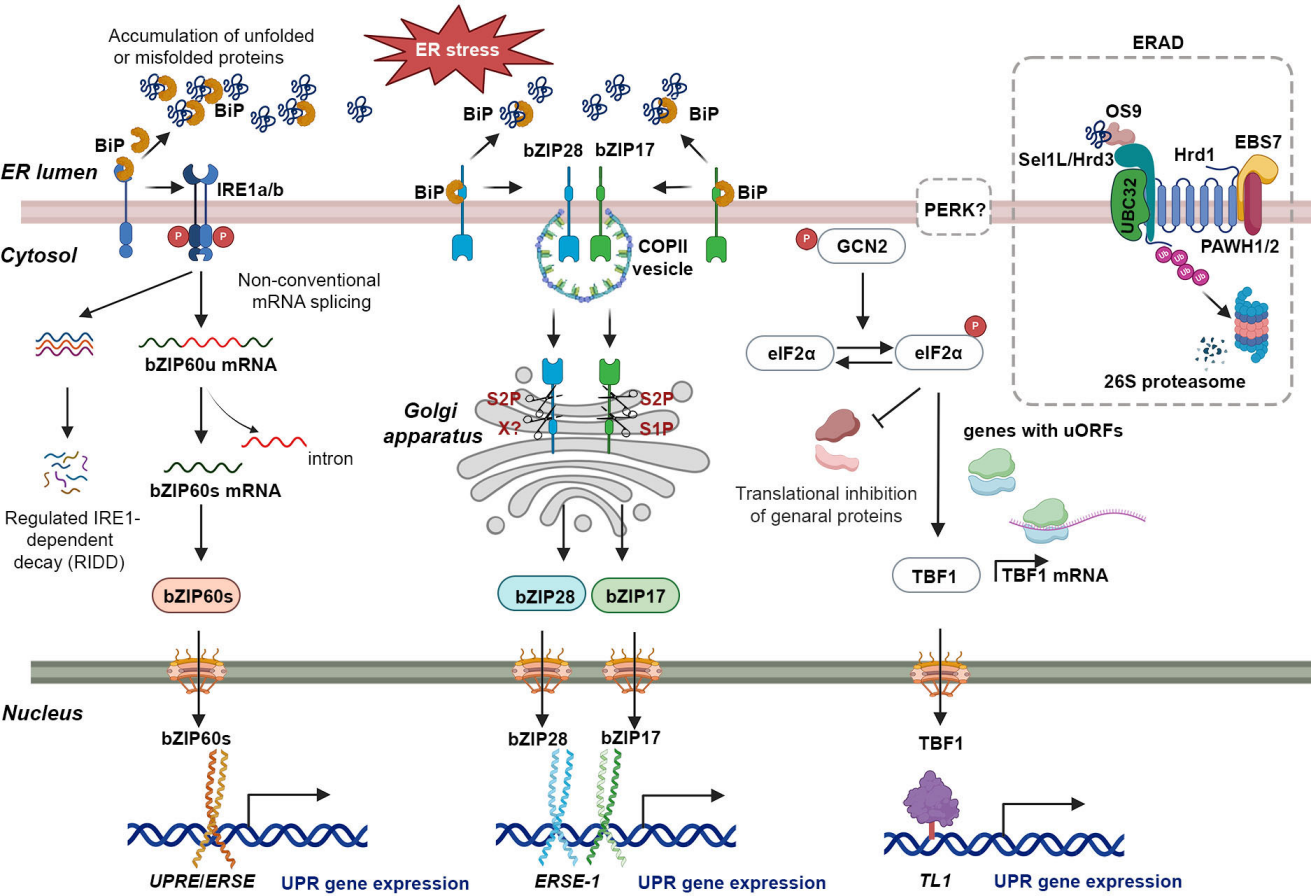
The UPR in plants: a signaling network coordinating ER homeostasis and stress adaptation. The UPR is activated by the accumulation of unfolded proteins in the ER due to various factors (top left of figure). BiP binds to unfolded proteins, leading to dissociation of IRE1a and IRE1b. Activated IRE1 then cleaves a specific intron from bZIP60u mRNA, generating bZIP60s mRNA. bZIP60s, a potent transcriptional activator, translocates to the nucleus and binds to UPREs and ERSEs in target gene promoters, inducing the expression of stress-responsive genes. In addition to its role in splicing bZIP60 mRNA, activated IRE1 is also involved in a process called Regulated IRE1-dependent decay (RIDD). Under conditions of chronic stress, IRE1 hyper-activates and cleaves additional mRNAs through RIDD. The bZIP17 and bZIP28 pathway is activated by ER stress in plants. Unfolded proteins bind to BiP, causing bZIP17 and bZIP28 to dissociate from the ER membrane. These transcription factors are transported to the Golgi, where proteolytic cleavage mediated by S1P and S2P enzymes releases their N-terminal domains. The N-terminal domains contain the necessary domains for their function as transcription factors. Upon translocation to the nucleus, they bind to ERSE-1 sequences in target gene promoters, inducing the expression of UPR-associated genes encoding ER chaperones and ERAD proteins involved in protein folding, quality control, and degradation within the ER. GCN2 is a kinase activated by dimerization and autophosphorylation in response to endoplasmic reticulum stress. It phosphorylates eIF2α, leading to widespread inhibition of mRNA translation. However, a specific group of uncapped mRNAs with upstream open reading frames (uORFs), such as TBF1 mRNA, are selectively translated. TBF1, a heat-shock factor-like transcription factor, binds to the *TL1 cis*-element, crucial for inducing BiP2 and CRT3. TBF1 also plays a role in coordinating developmental processes with stress responses, particularly in the growth-to-defense transition. During ER stress, ER chaperones assist in proper protein folding, while ERAD proteins eliminate irreversibly misfolded proteins. ERAD initiation involves OS9 recognizing the N-glycan on a misfolded protein and associating with Sel1L/Hrd3. The Hrd1-Sel1L/Hrd3-OS9 complex, along with UBC32, the E2 enzyme, promotes ubiquitination (Ub) of the misfolded protein for subsequent cytosolic degradation. This process helps restore ER homeostasis by removing unfolded proteins that could disrupt cellular functions.

The UPR constitutes a crucial regulatory process in plant cells, which is activated upon the presence of misfolded or unfolded proteins stress (Howell, 2021). Comprehensive investigations have been conducted to elucidate the UPR pathways in a range of plant species, encompassing maize, *Oryza sativa* (rice), *Solanum lycopersicum* (tomato), soybean, tobacco, and Arabidopsis (Lu et al., 2012; Czekus et al., 2020; Pastor-Cantizano et al., 2020; Czekus et al., 2022; Yang et al., 2022). In plants, the UPR is mediated through two distinct signal transduction pathways. The initial pathway, referred to as the IRE1 pathway, is facilitated by IRE1 and involves two isoforms in Arabidopsis, IRE1a and IRE1b (**Figure 1**) (Koizumi et al., 2001; Noh et al., 2002; Moreno et al., 2012). IRE1a and IRE1b possess homologous cytoplasmic regions characterized by the presence of a kinase domain, but exhibit functional divergence (Noh et al., 2002). IRE1a is primarily required for biotic stresses, while IRE1b plays a predominant role in abiotic stresses (Deng et al., 2011; Afrin et al., 2020). An additional isoform of IRE1, designated as IRE1C, has been identified as unique to plants (Mishiba et al., 2019). Recent evidence has shown that IRE1 promotes balanced cell expansion by restricting the Target of Rapamycin (TOR) kinase-dependent control of cellular differentiation (Angelos and Brandizzi, 2022). However, its precise role in the UPR remains to be elucidated. Under ER stress conditions, BiP binds to unfolded proteins, dissociating IRE1, which then undergoes trans-autophosphorylation and dimerization (**Figure 1**). The endonucleases IRE1a and IRE1b facilitate the removal of a 26-nucleotide intron from *bZIP60* mRNA, resulting in the production of the spliced variant *bZIP60s*, which encodes an active transcription factor (**Figure 1**) (Deng et al., 2011; Nagashima et al., 2011; Li et al., 2012; Moreno et al., 2012; Li et al., 2022). Upon activation, bZIP60s translocates to the nucleus, where it promotes the expression of genes associated with ER stress (**Figure 1**) (Iwata et al., 2008; Deng et al., 2011; Nagashima et al., 2011; Moreno et al., 2012; Hayashi et al., 2013; Sun et al., 2013; Xu et al., 2019). Under severe or prolonged ER stress, IRE1 also degrades many mRNAs on the ER membrane encoding secretory pathway proteins through a selective cleavage mechanism referred to as regulated IRE1-dependent decay (RIDD) (**Figure 1**) (Mishiba et al., 2013; Hayashi et al., 2016). The recent findings indicate that AtIRE1 determines cell fate during ER stress by balancing the UPR and the ubiquitin-proteasome system (UPS) via a key pro-death component, phosphatase type 2CA (PP2CA)-interacting finger protein 1 (PIR1). However, the mechanism by which AtIRE1 regulates PIR1 remains unknown (Ko et al., 2023).

The second pathway involves membrane-associated bZIP transcription factors bZIP17 and bZIP28, which are functional homologs of mammalian ATF6 (Liu et al., 2007a; Liu et al., 2007b). Under normal conditions, bZIP17/28 is retained in the ER due to its binding to the BiP protein (**Figure 1**) (Liu et al., 2007a; Srivastava et al., 2013; Srivastava et al., 2014; Henriquez-Valencia et al., 2015). Under ER stress conditions, bZIP17 and bZIP28 dissociate from BiP, become mobilized, and undergo translocation to the Golgi apparatus through coat protein complex II (COPII) vesicle-mediated transport (**Figure 1**) (Srivastava et al., 2012). In the Golgi apparatus, they undergo proteolytic processing by two resident site proteases, S1P and S2P, releasing their transcription factor (TF) domains (**Figure 1**) (Manghwar and Li, 2022). Subsequently, these TF domains translocate to the nucleus, where they act as transcription factors, enhancing the expression of ER stress-associated genes (**Figure 1**) (Liu et al., 2007b; Li et al., 2010; Liu et al., 2022). Nonetheless, a recent investigation has demonstrated that the activation of bZIP28 occurs through a sequential process involving S2P and as-yet-unidentified proteases, rather than S1P-mediated cleavage (Sun et al., 2015; Iwata et al., 2017). Both bZIP17 and bZIP28 can bind to ER stress response elements (ERSEs) and unfolded protein response elements (UPREs) at the promoter region of UPR-related genes, including BiPs (**Figure 1**) (Liu and Howell, 2010; Gao et al., 2022). Moreover, bZIP28 can interact with Nuclear transcription factor Y (NF-Y) and form a transcriptional complex to upregulate UPR-related genes (Liu and Howell, 2010). Typically, bZIP17 and bZIP28 exhibit comparable activation patterns in response to ER stress inducers, including chemicals like tunicamycin (TM) or dithiothreitol (DTT), cadmium (Cd) as well as environmental stresses such as heat stress and viral infections (Liu et al., 2007b; Li et al., 2010; Gao et al., 2022; Li et al., 2022; De Benedictis et al., 2023). However, they show differences in sensitivity in certain environmental stresses. For instance, under salt stress conditions, bZIP17 elevates the expression of the chaperone BiP3, whereas bZIP28 participates in responses to pathogen infections (Henriquez-Valencia et al., 2015; Qiang et al., 2021).

Although the PERK pathway, which is present in mammals, has not been identified in plants, General Control Non-repressible 2 (GCN2) has been identified as an orthologue of the elF2α kinase that responds to both abiotic and biotic stresses (Yu et al., 2022). Recently, Arabidopsis GCN2 was shown to activate the translation of a heat-shock factor-like transcription factor, TL1-binding transcription factor 1 (TBF1), which contains upstream open reading frames (uORFs) within its 5’ untranslated region (5’ UTR) (**Figure 1**) (Liu et al., 2019). This process is initiated in response to pathogen invasion, subsequently triggering specific transcriptional reprogramming through the expression of target genes (Lageix et al., 2008; Pajerowska-Mukhtar et al., 2012; Liu et al., 2019).

### Role in maintaining ER homeostasis and protein quality control

In situations of excessive or prolonged ER stress, where UPR mechanisms cannot restore protein folding, the ERAD system facilitates the clearance of terminally aberrant proteins, thus maintaining ER homeostasis (Vembar and Brodsky, 2008; Hwang and Qi, 2018). The ERAD comprises a multistep process, which includes the identification of cargo proteins, retro-translocation of substrates to the cytoplasm through an ER membrane channel, ubiquitination of ER proteins by ubiquitin enzymes, and subsequent degradation of ubiquitinated substrates via the 26S proteasome (**Figure 1**) (Chen et al., 2020). The ERAD machinery has been extensively studied in yeast and mammals (Ye et al., 2001; Sato et al., 2009; Avci and Lemberg, 2015; Habeck et al., 2015; Schoebel et al., 2017). In recent years, several ERAD components have been identified and characterized in plants (Chen et al., 2022). The N-glycans of misfolded proteins are recognized by osteosarcoma amplified 9 (OS9), which associates with the suppressor enhancer Lin12 1 like (Sel1L)/HMG-CoA reductase degradation protein 3 (Hrd3)/HMG-CoA reductase degradation 1 (Hrd1) complex (**Figure 1**) (Duwi Fanata et al., 2013). Protein associated with Hrd1-1/2 (PAWH1/2) interaction with EMS-mutagenized Bri1 suppressor 7 (EBS7) indirectly associates with Hrd1, regulating the stability and activity of the E3 ligase (**Figure 1**) (Liu et al., 2015; Lin et al., 2019). Therefore, Hrd1 has the potential to target UBC32, an E2 enzyme located on the ER membrane of Arabidopsis. UBC32 is responsible for the ubiquitination of aberrant proteins that is induced by stress, leading to their subsequent degradation in the cytosol via the proteasome pathway (**Figure 1**) (Cui et al., 2012; Chen et al., 2017; Chen et al., 2020).

ERAD, an important proteolytic pathway crucial to protein quality control, appears as a key factor in various studies associated with the enhancement of plant resistance to environmental stresses, productivity increase, and quality improvement. ERAD is a significant mechanism in plants for responding to environmental stresses, showing resistance capabilities to heat stress, drought, and salinity (Li et al., 2017; Strasser, 2018). In various plant species, evolutionarily conserved homologous ERAD components appear to be associated with stress tolerance and plant defense pathways elucidated two evolutionarily conserved ERAD pathways, DOA10 and HRD1, responding to heat stress in Arabidopsis (Liu and Li, 2014; Li et al., 2017; Strasser, 2018; Huber et al., 2021). This study demonstrated that loss-of-function mutants exhibited a higher survival rate and lower electrolyte leakage compared to the wild-type plants, enhancing plant resistance to heat stress (Li et al., 2017; Strasser, 2018). ERAD influences plant productivity by managing ER stress caused by protein misfolding. In this context, Ohta and Takaiwa (2015) showed that OsHrd3 is necessary for maintaining the quality of ER-derived protein bodies in rice endosperm (Ohta and Takaiwa, 2015). Additionally, Wakasa et al. (2011) proposed the possibility of improving the protein quality of rice through the role of ER stress response and ERAD (Wakasa et al., 2011). However, further research is necessary for a comprehensive understanding of ERAD associated with enhancing plant stress resistance, productivity, and quality.

### CRISPR-Cas system as a versatile genome editing tool in plants

**Figure 2:**
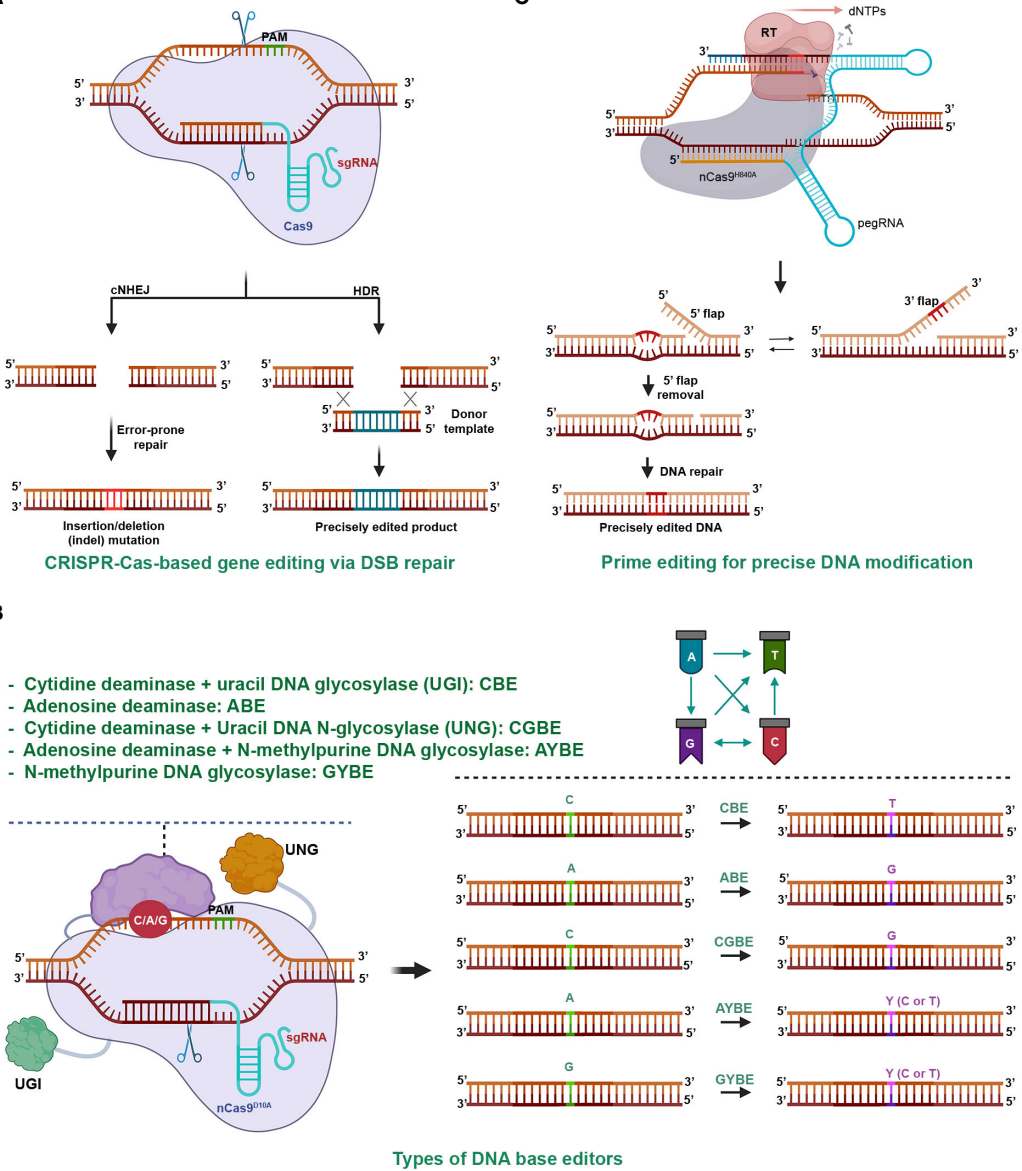
CRISPR-Cas-Mediated Gene Editing. **(A)** CRISPR-Cas9-Mediated Gene Editing through Double-Stranded Break (DSB) Repair. The CRISPR-Cas complex cleaves both strands of the target DNA, resulting in a DSB. The repair of the DSB predominantly occurs through two pathways: nonhomologous end joining (NHEJ) and homologous recombination (HR). NHEJ usually restores the original DNA sequence, but it can lead to imperfect repair and DNA insertion or deletion mutations, particularly during intense DSB formation. HR precisely inserts the desired sequence (green sticks) into the genomic region using a DNA donor template with homologous ends to the DSB terminals. **(B)** DNA base editors for genome editing. Base editing involves a deaminase, usually fused with a Cas9 (nCas9^D10A^) nickase, to remove an amino group from a nucleobase on the non-target strand. The deaminated base is then repaired via base excision or nucleotide excision repair, resulting in base transitions or transversions. Depending on the type of deaminase used, base conversion can lead to transitions, such as cytidine deaminase for C/G to A/T in cytosine base editors (CBE), or A/T to G/C in adenine base editors (ABE). Adding uracil DNA N-glycosylase inhibitors (UGI) enhances CBE efficiency. Base transversions can be achieved by adding uracil DNA N-glycosylase (UNG) to CBE (C/G to G/C in CGBE), N-methylpurine N-glycosylase to ABE (A/T to C/G or T/A), or by using UNG alone (G/C to C/G or T/A with GYBE). **(C)** Prime editing for precise DNA modification. The prime editing utilizes a pegRNA and a reverse transcriptase (RT) enzyme fused to the C-terminal of a nCas9^H840A^. It copies genetic information from the 3’ extension of the pegRNA into the nicked end on the non-target strand. By introducing desired genetic changes within the RT template of the 3’ extension, prime editing enables precise genetic modifications at the target site. Prime editing allows for a wide range of precise DNA changes within a genome, including various types of base conversion, DNA insertion, and deletion.

The CRISPR-Cas system is an adaptive immune mechanism used by bacteria to defend against the invasion of bacteriophages (Mojica et al., 2005). The system comprises an endonuclease (Cas) and a guide RNA (gRNA) that together form a ribonucleoprotein complex. The Cas complex locates and binds to a target dsDNA with the help of the guidance of gRNA. Once the complex is activated, the Cas enzyme cleaves the phosphodiester bonds of both strands, creating a double-stranded break (DSB) in the target DNA (Barrangou et al., 2007). The cells repair the DSB using nonhomologous end-joining (NHEJ) or homologous recombination (HR) (**Figure 2A**), which may result in mutations or modifications in the DNA sequence, thereby achieving gene editing (Jinek et al., 2012; Cong et al., 2013; Dickinson et al., 2013; Schwank et al., 2013; Van Vu et al., 2019).

Since its repurposing for customized DNA cleavages and subsequent gene editing in 2012, the CRISPR era has begun (Jinek et al., 2012). The technology, recognized for its potential in precise genome engineering, has proven valuable in agriculture and various other fields, paving the way for numerous applications and advancements (Chen et al., 2019). As of today, there are two classes, six types, and over 30 subtypes of the CRISPR-Cas system that function in DNA or RNA targeting or other activities (Koonin and Makarova, 2022). The system has not only been repurposed as molecular scissors but also for other applications such as transcriptional regulations (Cheng et al., 2013; Qi et al., 2013). Furthermore, the editing scope has expanded from single bases (**Figure 2B**) (Komor et al., 2016; Gaudelli et al., 2017; Chen et al., 2021; Kurt et al., 2021; Tong et al., 2023) to small DNA changes with prime editing (**Figure 2C**) (Anzalone et al., 2019; Lin et al., 2020) and microhomology-mediated precision short DNA replacement (Tien Van et al., 2022), extending to kilobase-gene targeting, thereby establishing it as a versatile tool for genome editing (Dickinson et al., 2013; Schwank et al., 2013; Chen et al., 2019; Vu et al., 2020b).

The CRISPR-Cas system has been extensively utilized in plant biology and crop engineering, with a wide range of applications developed for editing target genes in both monocot and dicot species (Chen et al., 2019; Zhu et al., 2020). These applications include simple edits with indel mutations as well as HR-based precise gene replacement (Feng et al., 2013; Li et al., 2013; Nekrasov et al., 2013; Chen et al., 2019; Vu et al., 2020b). Due to the versatility of the CRISPR system, it has emerged as a valuable tool for achieving high efficiency in gene editing in plants, proving to be a significant asset in advancing plant biology and crop improvement research (Chen et al., 2019; Zhu et al., 2020; Tien Van et al., 2022).

### Exploring the versatility of CRISPR-Cas system in UPR pathways

The CRISPR-Cas system is a versatile tool with numerous applications, including gene functionalization and regulation. Cas9 complexes act like molecular scissors and can theoretically cleave any genomic site of interest if a PAM motif is present. Previously, researchers relied on knockout lines generated by T-DNA insertion, random mutagenesis, or RNA interference (RNAi)-based downregulation lines to assess gene function (Howell, 2013; Pucker et al., 2021). However, these methods have limitations such as complex T-DNA integration events (Tamura et al., 2016) or residual gene activity in downregulated lines (Santillan Martinez et al., 2020), which lead to complicated analysis. The emergence of CRISPR-Cas technology has revolutionized the study of gene function in plants, as knockout lines generated by CRISPR-Cas tools are more precise and cleaner than traditional methods (Tien Van et al., 2022). The CRISPR-Cas system is highly efficient, customizable, simple, and cost-effective, making it an accessible tool for labs worldwide (Ahmadzadeh et al., 2019; Chen et al., 2019; He et al., 2023).

Although CRISPR-Cas has been used extensively for gene regulation and functionalization since its discovery in 2012, its application in studying ER stress response is relatively recent and began in 2019 (Mishiba et al., 2019; Liu et al., 2020). Even though a few CRISPR-Cas-related studies have been conducted in this field, there is still considerable potential for further functional exploration of genes involved in ER stress signaling using this technology (**Tables 1**, **2**). Early research using CRISPR-Cas to study ER stress response focused on the Arabidopsis genes Protein Associated with PAWH1 and PAWH2 (**Table 1**) (Lin et al., 2019). These genes are essential components of the ERAD pathway (Lin et al., 2019) and play vital roles in mitigating environmental stress such as salinity (Liu et al., 2011). The PAWH genes were induced by ER stress and contributed to the stabilization of the UPR sensing complexes mediated by the EBS7 and Hrd1 (Lin et al., 2019). Additionally, the IRE1-mediated RNA splicing of *AtbZIP60* is a critical aspect of the UPR pathway in ER stress responses (Yu et al., 2022). To investigate the role of IRE1b in the signaling arm, the sensor domain-coding region of the gene was deleted using dual gRNA CRISPR-Cas9 complexes in the *ire1a*/*c* mutant background (**Table 1**) (Mishiba et al., 2019). The IRE1b-edited lines exhibited similar effects on BiP3 and PR-4 transcription as the *ire1a*/*b* mutant lines and bZIP60 RNA splicing as the *ire1a*/*c* mutants. However, there was no evidence of growth defects or seed set reductions in the mutant lines (**Table 1**) (Mishiba et al., 2019).

**Table 1:** Recent studies related to the UPR that used CRISPR-Cas9 tools.

No.	Target gene	Plant species	Gene function	Impact	Reference
**1**	*PAWH1* and *PAWH2*	*Arabidopsis thaliana*	Plant-specific components of ERAD complex	The *pawh1pawh2* double mutants suppressed the dwarf phenotype of the corresponding *bri1-5*	(Lin et al., 2019)
**2**	*IRE1B*	*Arabidopsis thaliana*	Splicing of bZIP60-encoding mRNA	Deletion of the IRE1B’s sensor domain by CRISPR-Cas9 showed no growth defect and seed set reduction	(Mishiba et al., 2019)
**3**	*OsNTL3*	*Oryza sativa*	Regulation the expression of OsbZIP74 and other UPR related-genes involved under heat stress conditions	The *ntl3* mutant plants showed more sensitive phenotype to heat stress treatment	(Liu et al., 2020)
**4**	*IAN2*, *IAN3*, *IAN4*, *IAN5*, *IAN6*, *OsIAN1*, and *OsIAN2*	*Arabidopsis thaliana* and *Oryza sativa*	Regulation of the HSR, UPR, and cell death	The single mutants (*ian3*, *ian5*, *ian6*) and double mutants (*ian2ian3*, *ian4ian5*) showed less barren siliques along their main inflorescences than wild type	(Lu et al., 2021)
**5**	*Sec23* isoforms	*Physcomitrium patens*	Influencing ER to Golgi apparatus trafficking and secretion to the plasma membrane	The *sec23d* mutant showed smaller phenotype and fewer gametophores than the wild type, while the quintuple *sec23abcfg* mutant have no detectable growth defects	(Chang et al., 2022)
**6**	*OsHLP1*	*Oryza sativa*	OsHLP1 promotes disease resistance by compromising ER homeostasis when plants are infected by pathogens	The *oshlp1* mutant showed compromising blast disease resistance in rice	(Meng et al., 2022)
	*OsNTL6*		ER homeostasis in rice during infection of *Magnaporthe oryzae*	The *osntl6* mutant plants showed enhanced disease resistance compared with wild type plants	
**7**	*OsbZIP60*	*Oryza sativa*	OsbZIP60 regulates the formation of grain chalkiness in rice via UPR	The *osbzip60* mutant plants showed high grain chalkiness rate and white floury endosperm	(Yang et al., 2022)
	*OsbZIP50*		OsbZIP50 played an important role in the formation of grain chalkiness	The *osbzip50* mutant plants had high grain chalkiness rates	
**8**	*NOBIRO6/TAF12b*	*Arabidopsis thaliana*	NOBIRO6/TAF12b contributes to UPR-associated root growth control	The *nobiro6* mutant plants rescue the root growth defect characteristic of the *bzip17bzip28* double mutant	(Kim et al., 2022a)
**9**	*NbbZIP60*	*Nicotiana benthamiana*	Geminivirus satellite-encode βC1 activates UPR, induces bZIP60 nuclear export, and manipulates the expression of bZIP60 downstream genes to benefit virus infection	The *nbbzip60* mutant plants showed milder curling symptoms than the wild type plants after inoculating these plants with TYLCCNV/TYLCCNB through agro-infiltration.	(Zhang et al., 2023)

**Table 2:** Recent promoter editing studies that used CRISPR-Cas9 tools.

No.	Target gene	Plant species	Promoter edited regions	Impact	Reference
**1**	*OsRAV2*	*Oryza sativa*	Mutation in the *GT-1* element regions of *OsRAV2*	Induced salt stress response	(Duan et al., 2016)
**2**	*ARGOS8*	*Maize*	Mutation of negative maize GOS2 promoter of *ARGOS8*	Enhanced drought stress conditions in the field	(Shi et al., 2017)
**3**	*SWEET11/13/14*	*Oryza sativa*	Mutation in the *EBEs* in the *SWEET11/13/14* promoters	Improved *Xanthomonas oryzae* pv. *Oryzae* resistance	(Oliva et al., 2019)
**4**	*LsGGP2*	*Lactuca sativa*	Mutation in the uORFs of *LsGGP2*	Increased tolerance to oxidative stress and ascorbate content	(Zhang et al., 2018)
**5**	*AtTBF1*	*Arabidopsis thaliana*	Strategy of mutation in the uORFs of *AtTBF1*	Expectation in response to pathogen attack	(Vuong et al., 2023)

In rice, the IRE1-mediated RNA splicing in ER stress responses is also conserved, and the OsbZIP74 (also known as OsbZIP50) is the ortholog of AtbZIP60 (Lu et al., 2012). Modified OsbZIP74 is transported to the nucleus and upregulates UPR-related genes, including several membrane-associated NAC transcription factors. In a recent study, researchers utilized the CRISPR-Cas9 gene-editing system to generate knockout mutants of OsNTL3, confirming its role as a transcriptional activator of OsbZIP74 (**Table 1**) (Liu et al., 2020). The investigation also showed that OsbZIP74 positively regulates the transcription of OsNTL3 under heat stress conditions. Additionally, the study revealed that the loss of OsNTL3 function results in increased heat sensitivity in rice seedlings (**Table 1**) (Liu et al., 2020). *OsbZIP60* has been identified as a critical regulator of grain chalkiness, a stress-related phenotype in rice (Hayashi et al., 2012; Takahashi et al., 2012; Yang et al., 2022). To understand the role of *OsbZIP60* in managing this response, *osbzip60* knockout mutants were created using CRISPR-Cas9 gene-editing technology. In these mutants, upregulation of several chaperone genes, including *OsbZIP50*, *OsBiP1*, *OsBiP2*, and *OsBiP3*, was observed, leading to varying degrees of grain chalkiness. This result indicates that *OsbZIP60* plays a critical role in regulating rice grain chalkiness and maintaining ER homeostasis (**Table 1**) (Yang et al., 2022). In another study, the roles of two bZIP transcription factors, bZIP17 and bZIP28, in the UPR pathway were explored (Liu et al., 2007a; Liu et al., 2007b). The *bzip17bzip28* double mutant displayed stress-related phenotypes, including severe dwarfism, low germination rate, and short roots, compared to the wild-type plant (Kim et al., 2018). To unravel the underlying mechanisms associated with the observed stress-related phenotypes, a suppressor mutant named *nobiro6* was created within the *bzip17bzip28* background using CRISPR-Cas9 technology. This triple mutant, *bzip17bzip28nbr6*, demonstrated a partial rescue of root growth, highlighting the role of NOBIRO6/TAF12b as a transcription cofactor in UPR-associated root growth control (**Table 1**) (Kim et al., 2022b). In a recent study aiming to understand the role of NbbZIP60 in plant defense responses to pathogens, *nbbzip60* knockout mutants were generated using CRISPR/Cas9-based technology (Zhang et al., 2023). These knockout mutant plants showed a reduced amount of viral DNA, leading to milder leaf curling symptoms compared to wild-type plants under virus infection (**Table 1**). These findings highlight how CRISPR-Cas9 technology is enabling a deeper understanding of UPR in plants, opening avenues for enhancing plant stress resistance.

### CRISPR-Cas mediated dissection of ER stress and plant responses to biotic stresses

The CRISPR-Cas system offers a significant advantage in generating multiple gene mutations through multiplexing methods with multiple gRNAs. For instance, it was used to generate various mutated variants in single and combinations of UPR-related genes encoding for immune-associated nucleotide-binding (IAN) proteins in Arabidopsis (**Table 1**) (Lu et al., 2021). In the study, IAN2 to IAN6 were found to be located at a single locus on chromosome 1 by genome-wide association study (GWAS) (Lu et al., 2021). The efficient CRISPR-Cas9 system was used to create single, double, triple, and quadruple IAN gene mutations that were crucial for gene functionalization. Interestingly, the *ian* mutants, particularly the *ian6* knockout lines, exhibited enhanced heat tolerance during the reproductive stage in both Arabidopsis and rice (**Table 1**) (Lu et al., 2021). The IAN6 protein has been discovered to localize to the ER, where it suppresses HSP and UPR-related gene expression and promotes programmed cell death during the reproductive stage (**Table 1**) (Lu et al., 2021). In a similar manner, multiplexed editing has been effectively used to generate combined knockout mutations in genes associated with COPII-mediated vesicle trafficking from the ER to the Golgi apparatus (Chang et al., 2022). The COPII complex is involved in transporting bZIP28 proteins to the Golgi for processing and subsequent release into the cytosol during ER stress (Srivastava et al., 2012; Howell, 2013). In plants, Sec23 and Sec24 combine with Sar1 to form the inner layer of COPII vesicles. Several isoforms of the Sec23 were shown to form distinct ER exit sites with differential effects on protein trafficking and growth (Yoshihisa et al., 1993; Zeng et al., 2015). The *sec23d* mutant significantly hindered ER-to-Golgi transport, whereas the quintuple *sec23abcfg* mutant primarily impacted protein secretion to the plasma membrane (**Table 1**) (Chang et al., 2022).

Plant responses to biotic stress, such as those induced by phytopathogens, require the maintenance of ER homeostasis. In rice, OsHLP1, which is induced by Magnaporthe oryzae infection, has been shown to interact with OsNTL6. This interaction suppresses the accumulation of OsNTL6, leading to the activation of genes involved in plant immunity and resulting in enhanced disease resistance (Meng et al., 2022). CRISPR-Cas9 knockout mutants of OsHLP1 displayed reduced disease resistance, suggesting that OsHLP1 positively regulates blast resistance in rice (**Table 1**) (Meng et al., 2022). In contrast, the OsNTL6 protein acts as a negative regulator of blast disease resistance; overexpression lines led to increased *Magnaporthe oryzae* infection, while knockout lines generated using CRISPR-Cas9 showed reduced infection compared to wild-type plants (**Table 1**) (Meng et al., 2022). Recent application of CRISPR-Cas technology in studying ER stress and plant responses to biotic stress has provided valuable insights into critical components of these pathways (Lin et al., 2019; Mishiba et al., 2019; Liu et al., 2020; Lu et al., 2021; Chang et al., 2022; Meng et al., 2022). By using CRISPR-Cas to edit the genome of plants, researchers can generate mutations in UPR-related genes to study their function and determine their role in the UPR pathway. Overall, the CRISPR-Cas system has revolutionized the field of genetics and has significant implications for the study of gene function in plants, including UPR-related genes.

### Enhancing stress tolerance and crop improvement through the CRISPR-Cas mediated modification of *cis*-regulatory elements

**Figure 3:**
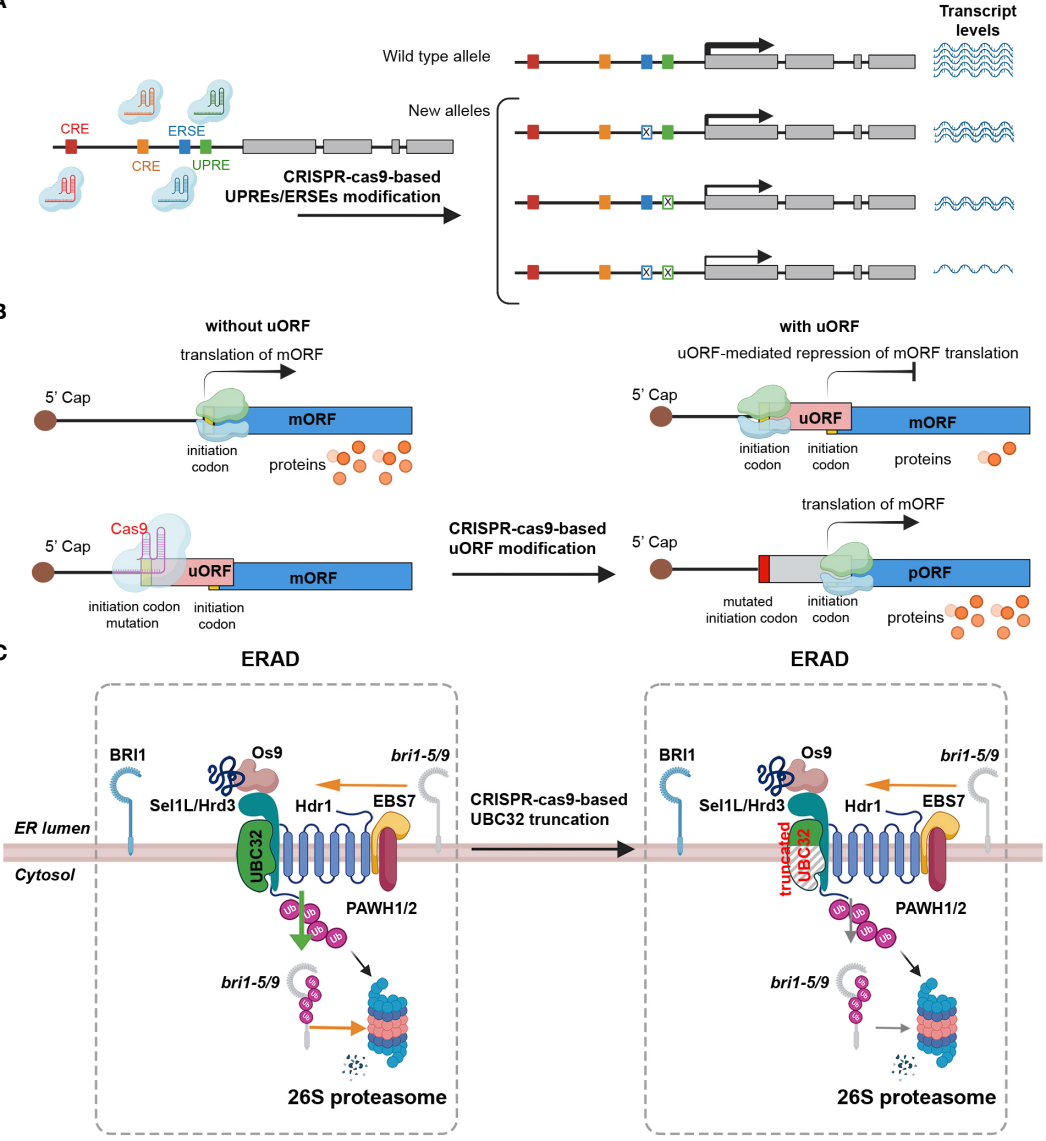
Strategies for prospective editing of ER stress signaling components to enhance stress tolerance. **(A)** CRISPR-Cas9 can be effectively employed to edit the promoter region of target genes in order to modulate gene expression. The promoter region contains important *cis*-regulatory elements (CRE, brown and ocher boxes), endoplasmic reticulum stress response elements (ERSEs, blue boxes), and unfolded protein response elements (UPREs, green box). These elements act as enhancers or repressors, playing a crucial role in regulating the transcriptional activity of the gene. By utilizing a multiplex genome editing approach, multiple single-guide RNAs (sgRNAs) can be designed to specifically target distinct ERSEs and UPREs within the promoter region. The CRISPR-Cas9 system, guided by these sgRNAs, induces double-strand breaks at the desired sites in the promoter region, leading to DNA repair mechanisms that can introduce stochastic mutations. These stochastic mutations occurring in the promoter region lead to the generation of alleles with diverse patterns and levels of gene expression. Certain mutations may enhance gene expression, while others may repress it. Implementing this method has the potential to generate a spectrum of phenotypic variations across different lines. **(B)** CRISPR-Cas9 can be used to manipulate gene translation by targeting upstream open reading frames (uORFs). By utilizing the CRISPR-Cas9 system, specific mutations can be introduced into the start codon region of uORFs, disrupting their inhibitory effects on translation. The translation process of messenger RNA (mRNA) begins when small (light blue) and large (light green) ribosomal subunits scan the mRNA from its 5′ cap (represented by a dark brown circle). The initiation codon, represented by a yellow box, serves as the starting point for translation. However, if the mRNA contains an upstream open reading frame (uORF) represented by a pink rectangle, the ribosome can stall at the uORF. This stalling event leads to the repression of translation of the main open reading frame (mORF) indicated by a blue rectangle. Consequently, the reduced translation of the mORF results in a decreased production of protein products, represented by orange circles. The mutated initiation codon (red rectangle) within uORF regions using the CRISPR-Cas9 inhibits ribosome stalling, resulting in increased production of proteins encoded by the mORF. **(C)** Strategy to generate truncated UBC32 using CRISPR-Cas9-mediated knockout to enhance BR signaling by stabilizing structurally imperfect, yet biochemically active, bri1 peptides to achieve stress tolerance. The strategy to enhance brassinosteroid (BR) signaling and improve stress tolerance involves the generation of a truncated form of the *ubiquitin-conjugating enzyme 32* (*UBC32*) gene using CRISPR-Cas9-mediated knockout. *UBC32* is responsible for encoding an E2 ubiquitin-conjugating enzyme that plays a crucial role in the degradation of the biochemically active but structurally incomplete brassinosteroid insensitive 1 (*bri1*: *bri1-5* or *bri1-9*) peptide. Through the process of ubiquitination, UBC32 targets the *bri1-5* or *bri1-9* peptide for 26S proteasome-mediated degradation in the cytosol. However, utilizing the CRISPR-Cas9 to disrupt UBC32 allows for a reduction in the ubiquitination of *bri1-5* or *bri1-9* peptide, leading to increased stability of the peptide. This enhanced stability contributes to the amplification of BR signaling, thereby improving stress tolerance in plants.

*Cis*-regulatory elements (CREs) are noncoding DNA sequences that incorporate transcription factors and other molecular binding sites, such as promoters and enhancers, which influence transcription (Wittkopp and Kalay, 2011; Wolter et al., 2019). The promoter regions of most plant UPR-related genes contain a consensus *cis*-acting element known as the ERSE and/or the UPRE (Liu and Howell, 2010; Howell, 2013; Nawkar et al., 2017). Several studies have underscored the significant potential of crop improvement through the editing of regulatory sequences to adjust gene expression levels, thereby generating novel phenotypic variants (Wolter and Puchta, 2018; Wolter et al., 2019). For instance, the *RAV2* gene, which is transcriptionally induced by salt stress in rice, was subjected to CRISPR-Cas-mediated engineering to modify the *GT-1* element in the promoter, revealing that the *GT-1* element directly governs the salt stress response (**Table 2**) (Duan et al., 2016; Li et al., 2020b). In maize, ARGOS8, a negative regulator of ethylene responses, enhances drought tolerance (**Table 2**) (Shi et al., 2015). Plants edited with CRISPR-Cas9 to replace the native promoter region of the *ARGOS8* gene with the *GOS2* promoter demonstrated increased grain yield under drought stress conditions in the field (**Table 2**) (Shi et al., 2015; Shi et al., 2017; Wang et al., 2022). Similarly, the removal of a regulatory fragment containing a transcription-activator-like effector (TALe)-Binding Element (EBE) in the promoter of *SWEET11* via CRISPR/Cas resulted in improved disease resistance in rice, without affecting fertility (**Table 2**) (Li et al., 2020a). This development presents a clear advantage over the sterile phenotype of the *Ossweet11* knockout mutant, which is unsuitable for crop improvement. Recently, genome editing of EBEs in *SWEET* promoter genes led to broad-spectrum bacterial blight resistance in rice (**Table 2**) (Oliva et al., 2019). While predicting the effects of gene expression regulation by manipulation of various CREs, including ERSEs and UPREs, and the resulting phenotypic changes could be difficult, the modification of CREs by CRISPR-Cas holds the potential to be a critical strategy not only for studying UPR signaling pathways, but also for breeding plants with stress tolerance and desirable traits (**Figure 3A**).

### Upstream open reading frames as regulatory elements and CRISPR-Cas9 applications for crop improvement

The uORFs are essential regulatory elements located in the 5’ UTR of main open reading frames (mORFs). Recent bioinformatics analyses estimate that approximately 35% of total plant transcripts contain uORFs (Silva et al., 2019; Li et al., 2021). These uORFs are known to act as inhibitors, repressing the initiation of mORF translation via ribosome stalling (Silva et al., 2019; Zhang et al., 2020). A recent research indicates that uORFs possess the ability to regulate gene expression in response to environmental stresses, as they control specific master regulators involved in stress responses (Zhang et al., 2020). Under adverse environmental conditions, stress-responsive transcripts containing uORFs are upregulated, suggesting that CRISPR-Cas9-mediated uORF editing could be a promising approach to enhance gene expression for crop trait improvement (**Figure 3B**) (Um et al., 2021). For example, the application of CRISPR-Cas9 editing to the uORF of LsGGP2 in *Lactuca sativa* (lettuce) has yielded promising results, demonstrating increased tolerance to oxidative stress and a substantial 150% increase in ascorbate content (**Table 2** and **Figure 3B**) (Zhang et al., 2018). Similarly, in Arabidopsis, TBF1 plays a critical role in the growth-to-defense transition in response to pathogen attack (**Table 2**) (Traubenik et al., 2021). Under normal conditions, two uORFs in TBF1 inhibit AtTBF1 translation; however, upon pathogen infection, these inhibitory effects are relieved, allowing TBF1 to regulate and induce the expression of defense-related genes (**Table 2** and **Figure 3B**) (Pajerowska-Mukhtar et al., 2012). These findings suggest that CRISPR-Cas-based gene editing can be used to remove or generate uORF sequences in target genes. This strategy can be used to increase or decrease protein translation levels, and applied to develop crops with improved traits, including stress resistance (Vuong et al., 2023). In conclusion, CRISPR-Cas9-mediated uORF editing represents a promising avenue for enhancing plant resilience to environmental stresses and advancing crop trait improvement.

## Strategies for enhancing ER stress tolerance in plants using CRISPR-Cas9

Regulating plant responses to ER stress is essential for enhancing crop productivity and survival rates. To address this gap, we propose several strategies for using CRISPR-Cas tools to enhance ER stress tolerance in plants. One approach involves editing genes that directly or indirectly regulate the UPR signaling pathway. However, it is important to note that a thorough understanding of the roles of the targeted genes is necessary for designing an effective editing strategy. The most straightforward method for breeding ER stress tolerance using CRISPR-Cas complexes involves introducing indel mutations into the coding sequences of targeted genes and selecting knockout lines. The fundamental concept behind employing simple indel mutations to improve crop performance is to target genes known to regulate a specific response or trait. This method has been proven to be effective in improving crop performance (Chen et al., 2019; Vu et al., 2020a; Zhu et al., 2020). However, it is necessary to ensure that the targeted knockout mutants have minimal negative impact on plant morphology, agronomical traits, growth, development, and yield. A minimal trade-off in growth, development, and yield may be acceptable if the benefits to other agronomic traits are highly significant. Genes that directly or indirectly regulate the UPR signaling pathway could be targets for gene editing to confer ER stress tolerance in plants. However, fundamental studies are necessary to identify the specific genes that are suitable for the editing approach, and it is crucial to ensure that the benefits outweigh any potential trade-offs.

UPR regulators play a crucial role in maintaining ER homeostasis in both normal and stress conditions. Identifying genes involved in ERAD pathways is of great importance. One such gene is *UBC32*, which is involved in the ERAD-mediated quality control process through ubiquitination-associated protein degradation. The mutation of *UBC32* results in the accumulation of structurally abnormal *bri1-5* and *bri1-9* mutant forms of brassinosteroid insensitive 1 (BRI1). Despite their structural abnormalities, these mutant forms still retain the biochemical activity of the BRI1 receptor, subsequently enhancing brassinosteroid (BR) signaling (Cui et al., 2012). The *ubc32* single and *bri1-5/9 ubc32* double mutant lines shows improved salt stress tolerance compared to the wild-type control (**Figure 3C**) (Cui et al., 2012; Zhou et al., 2021). Interestingly, the *ubc32* single mutant Arabidopsis demonstrates a phenotype similar to the wild-type (WT) Col-0 when grown under normal conditions (**Figure 3C**) (Zhou et al., 2021). These findings suggest that *UBC32* could be a promising target for enhancing stress tolerance via CRISPR-Cas9-mediated knockout. By employing gRNAs to direct CRISPR-Cas9, cleavage of the UBC32 coding sequence can be achieved. This results in indel mutations that cause premature termination of translation and truncation of the UBC32 polypeptide chain, which may ultimately enhance salt tolerance by affecting BR signaling.

### Enhancing plant stress tolerance through CRISPR-Cas editing of key regulatory genes

Plant growth and development are significantly influenced by environmental conditions such as light (Bae and Choi, 2008; Kami et al., 2010; Paik and Huq, 2019). Suboptimal lighting conditions can trigger ER stress responses, which can lead to cellular dysfunction and ultimately affect plant growth and survival (Mawphlang and Kharshiing, 2017; Ahn et al., 2022). The connection between light and UPR has been shown to be mediated by ELONGATED HYPOCOTYL 5 (HY5), a bZIP factor previously known as a master regulator of light signaling (Gangappa and Botto, 2016). HY5 acts as an important transcription factor in both light signaling and the UPR pathway. In the dark, HY5 is targeted for degradation by the E3 ubiquitin ligase COP1, which marks HY5 with ubiquitin and targets it for degradation by the 26S proteasome (Ang et al., 1998; Xu et al., 2016). However, in the presence of light or under ER stress conditions, HY5 is stabilized and can regulate the expression of UPR-related genes (Nawkar et al., 2017). The *HY5* gene has been identified as a critical regulator of stress resistance in plants (Xiao et al., 2021). Therefore, the utilization of CRISPR-Cas-based gene editing technology for the generation and characterization of crops carrying HY5 alleles may present a promising and compelling direction for further scientific investigation. Under various stress conditions, the levels and activity of HY5 protein increase, leading to enhanced expression of downstream genes. Overexpression or complete removal of the *HY5* gene may result in significant trade-off in the phenotypes of edited lines, due to the important role of HY5 in multiple processes, such as photomorphogenesis. Consequently, generating mutants with HY5 alleles that maintain a certain level of expression could be an interesting approach for developing crops that efficiently respond to various stresses. The CRISPR-Cas-mediated generation of crops with HY5 alleles could be achieved by targeting the *cis*-regulatory elements of the *HY5* gene. This approach has already been shown to be effective in previous studies (Rodriguez-Leal et al., 2017). Additionally, introducing CRISPR-Cas-mediated precise modification of the DNA binding bZIP domain or the COP1 binding domain in HY5 may be alternative strategies to alter HY5 functions. Such a strategy could be readily implemented through CRISPR-Cas-based gene targeting or prime editing (Van Vu et al., 2019). In summary, the regulatory function of HY5 in various stress responses makes it a promising target for enhancing stress tolerance in plants via CRISPR-Cas-based gene editing. By generating mutant alleles of HY5, it might be possible to indirectly alter the expression of downstream genes, including UPR-related genes, and consequently enhance the resistance of plants to various stresses.

Cadmium, a hazardous heavy metal, significantly impacts plants by interfering with crucial processes such as water and nutrient uptake, photosynthesis, calcium signaling, and genome maintenance. This interference leads to stunted growth, diminished yield, and in severe cases, plant death (Xi et al., 2016; De Benedictis et al., 2023). Recent studies have demonstrated that knockout of the supernumerary aleurone 1 (SAL1) enzyme can mitigate the toxicity of cadmium in Arabidopsis plants (Xi et al., 2016). SAL1, also known as FIERY1, is a well-established regulator of stress response signaling. This enzyme possesses 3’(2’),5’-bisphosphate nucleotidase and inositol polyphosphate 1-phosphatase functions (Quintero et al., 1996; Xiong et al., 2001; Wilson et al., 2009), and is implicated in leaf morphogenesis (Robles et al., 2010). These findings suggest that targeting SAL1 using CRISPR-Cas-based techniques could be a promising approach to alleviating cadmium toxicity and other environmental stresses in crop plants. By knocking out SAL1, plants may be better equipped to cope with environmental stresses, leading to improved crop yields and sustainability. In conclusion, the recent discovery of the role of SAL1 in mitigating cadmium-induced toxicity and ER stress responses offers an exciting opportunity for enhancing the sustainability of crop production. By utilizing CRISPR-Cas-based techniques to target SAL1 and other regulators of ER stress responses, crop plants may exhibit increased resilience to environmental stresses, ultimately leading to enhanced crop yield and quality. Taken together, the proposed strategies for improving crop stress tolerance through genetic modification present a promising opportunity for enhancing plant tolerance and agricultural productivity. By targeting key genes involved in stress responses, such as *UBC32*, *HY5*, and *SAL1*, it may be possible to develop crops that are better adapted to challenging environmental conditions. However, further research is necessary to identify the optimal genes for modification and ensure that the benefits outweigh any potential drawbacks.

## Concluding remarks

The UPR mechanism plays a vital role in facilitating the growth and survival of plants under unfavorable environmental conditions (**Figure 1**). Despite the extensive research on the molecular mechanism of plant UPR, the adoption of CRISPR-Cas-based gene editing technology has been slow. This has limited the potential for developing crops with resistance to a variety of adverse biological and non-biological environmental conditions, including ER stress. Consequently, it is imperative to actively consider the use of CRISPR-Cas technology to study the functions of genes involved in ER stress responses and expand the scope of plant gene editing (**Figure 2**). Understanding how UPR is activated and regulated, as well as the consequences of such regulation, can provide valuable insights into the development of crops with resistance to various stresses. As plants continue to face unpredictable environmental stresses that can significantly impact crop yield and quality, the adoption of CRISPR-Cas-based gene knockout of UPR-related genes is of great importance. In this review, we summarize the current understanding of ER stress signaling and regulation, as well as the recent advances in CRISPR-Cas technology for ER stress research (**Tables 1**, **2**). Additionally, we discuss the prospects of using CRISPR-Cas-based gene editing for crop breeding, particularly in the development of crop varieties with enhanced ER stress tolerance (**Figure 3**). We hope that our review will help expand research in this field and attract attention to the potential of CRISPR-Cas technology for studying gene functions related to ER stress responses and expanding the scope of plant gene editing. In this review, we summarize the current understanding of ER stress signaling and regulation, as well as the recent progress made in CRISPR-Cas technology for ER stress studies (**Tables 1**, **2**). We also discuss the future prospects of using CRISPR-Cas-based gene editing for crop breeding, particularly in the development of crop varieties with enhanced stress tolerance We hope that our review will help propel the field and attract attention to the potential of CRISPR-Cas technology for studying gene functions related to ER stress responses.

## Author contributions

BV: Conceptualization, Methodology, Writing – original draft, Writing – review & editing. TV: Conceptualization, Funding acquisition, Methodology, Supervision, Writing – original draft, Writing – review & editing. JY: Writing – original draft. NN: Writing – original draft. KK: Writing – original draft. J-YK: Conceptualization, Funding acquisition, Methodology, Supervision, Writing – original draft, Writing – review & editing. KL: Conceptualization, Funding acquisition, Methodology, Supervision, Writing – original draft, Writing – review & editing.

## Glossary

**Table:**

ATF6	activating transcription factor 6
BiP3	luminal-binding protein 3
BR	brassinosteroid
BRI1	brassinosteroid insensitive 1
bZIP	basic leucine zipper protein
Cd	cadmium
COPII	coat protein complex II
CREs	*cis*-regulatory elements
Hrd1	HMG-CoA reductase degradation protein 1
DSB	double-stranded break
DTT	dithiothreitol
EBS7	EMS-mutagenized Bri1 suppressor 7
ER	endoplasmic reticulum
ERAD	ER-associated protein degradation
ERSE	ER stress response element
GCN2	general control non-repressible 2
gRNA	guide RNA
GWAS	genome-wide association study
HR	homologous recombination
Hrd1	HMG-CoA reductase degradation protein 1
Hrd1	HMG-CoA reductase degradation 3
HSP	heat shock protein
IRE1	inositol requiring enzyme 1
IAN	immune-associated nucleotide-binding
HY5	elongated hypocotyl 5
mORFs	main open reading frames
NHEJ	nonhomologous end-joining
NBT	new breeding techniques
NF-Y	Nuclear transcription factor Y
OS9	osteosarcoma amplified 9
PAWH1	protein associated with Hrd1-1
PERK	double-stranded RNA-activated protein kinase (PKR)-like endoplasmic reticulum kinase
PIR1	phosphatase type 2CA (PP2CA)-interacting finger protein 1
RIDD	regulated IRE1-dependent decay
RNAi	RNA interference
SAL1	supernumerary aleurone 1
Sel1L	suppressor enhancer Lin12 1 like
S1P	site-1 proteases
TBF1	TL1-binding transcription factor 1
TF	transcription factor
TOR	Target of rapamycin
TM	tunicamycin
UBC32	ubiquitin-conjugating enzyme 32
uORFs	upstream open reading frames
UPR	unfolded protein response
UPRE	unfolded protein response element
UPS	ubiquitin-proteasome system
UTR	untranslated region
XBP1.	X-box binding protein 1
